# Howard Malmstadt: an inspiration

**DOI:** 10.1155/S1463924688000422

**Published:** 1988


					Journal of Automatic Chemistry Vol. 10, No. 4 (October-December 1988), pp. 198-200
Howard Malmstadt: an inspiration
Thefollowing article appeared in "Analytical Chemistry', Vol. 60,
No. 2. Honouring, as it does, one of Journal of Automatic
Chemistry's' editorial advisers, the editor felt it important to
reprint it. The editor and publisher gratefully acknowledge the
permission of the American Chemical Society to reproduce the
article.
At the annual meeting of the Federation of Analytical
Chemistry and Spectroscopy Societies (FACSS), which
took place in Detroit, 4 to 9 October 1987, Howard V.
Malmstadt was doubly honoured. Malmstadt, professor
emeritus at the University of Illinois and provost and
senior vice-president at the Pacific and Asia Christian
University, accepted the 1987 ANACHEM Award. In
addition, more than 50 chemists, all academic progeny of
Malmstadt, contributed some 150 technical papers to the
conference.
The ANACHEM Award, which is given annually by the
Association of Analytical Chemists, honours Malm-
stadt's outstanding contributions to analytical research
and education. The technical presentations by Malm-
stadt's former students (and their students) convincingly
demonstrated the wide-ranging influence and importance
of the man in both areas.
Howard Malmstadt was born in Marionette, Wisconsin,
USA, in 1922. He earned his bachelor's degree at the
University of Wisconsin in 1943. During a subsequent
three-year hitch in the Navy, he studied electronics at
Princeton and at the Massachusetts Institute ofTechnol-
ogy and served as a radar officer in the Pacific. After the
war he returned to the University ofWisconsin, where he
obtained his M.S. (1948) and Ph.D. (1950) degrees in
analytical chemistry. In 1951 Malmstadt began a long
tenure at the University of Illinois, during which he
taught, performed research, and authored or coauthored
more than 150 technical articles and 10 books. He retired
from Illinois in the early 1980s and assumed his current
position at the Pacific and Asia Christian University in
Kailua-kona, Hawaii.
During his years at the University of Illinois, Malmstadt
earned a reputation as an inspirational force in the
analytical community. Gary Hieftje, an early student of
Malmstadt's, now at Indiana University, recently reflec-
ted: 'He [Malmstadt] emphasized setting high goals. He
would urge his students, by example, to set up standards
or ideal characteristics that they would like an instrument
to possess. He presented an ideal framework for academic
growth. And there is tremendous success among his
former students'.
Jim Winefordner, the first of Malmstadt's students to
enter academia, and now at the University of Florida,
echoed Hietje: 'Possibly he [Malmstadt] produced more
1988 American Chemical Society.
Ph.D.s that pursued academic careers than did any other
professor. I think it was something like 20. In terms of
scientific ability, he was brilliant. He always had good
suggestions, even though he covered a wide swath of
projects'. Hieftje concurred: 'His students studied ever-
thing and went in every direction in analytical chemistry'.
Chris Enke, now at Michigan State, has collaborated
with Malmstadt on many projects. Enke recently stated,
'Instead ofjust speaking ofprinciples and ideals, Howard
has lived them'. If one area stands out as being strongly
advocated and advanced by Malmstadt, it is electronics.
He was an innovator and a leader in the development of
modular electronic instruments, and he developed a
popular and influential course in electronics for scientists.
Stan Crouch, another early student, now at Michigan
State, summed it up in this way: 'He [Malmstadt]
introduced electronics to chemists. He had the vision to
see that chemists would be using electronic tools. It is
now taken for granted- thanks to Malmstadt- that an
analytical chemist will know electronics'. Malmstadt's
interest in electronics resulted in the 1962 book, Electronics
for Scientists, coauthored by Enke and E. C. Toren, Jr.,
and the 1981 book, Electronics and Instrumentation for
Scientists, coauthored by Enke and Crouch.
Malmstadt is also known for his efforts in the area of
kinetic methods. 'He promoted and fostered these
methods', said Crouch. Another area that has benefited
from Malmstadt's influence is spectroscopy. 'People
forget the spectroscopy part', opined Crouch, 'I am
creating a spectroscopy text that owes very much to
Howard Malmstadt and his course in spectroscopy [that
is, the one he developed at the University of Illinois]'.
Malmstadt's professional achievements and past honours
are many. He is a former Guggenheim Fellow and was the
chairman of the ACS Division of Analytical Chemistry.
He has been on the advisory boards of the National
Science Foundation, the National Institutes of Health,
the National Research Council, and various scientific
journals. He has served as a consultant for US industries
and the United Nations and has been a visiting professor
or guest lecturer at universities and scientific societies in
the USA, UK, Australia, Brazil, Singapore, India,
Japan, and China.
Malmstadt's many awards include the following: the
Instrument Society ofAmerica Award, the ACS Awards
in Analytical Chemistry and Chemical Instrumentation,
the Pittsburgh Conference Outstanding Chemist Award,
the Fulbright-Hay Distinguished Professor Award, the
Isco Award for Contributions in Biochemical Instrumen-
tation, and the ACS Division of Analytical Chemistry
Excellence in Teaching Award.
In bringing together so many academic relations in one
198
Howard Malmstadt
The Malmstadt family tree ofPh.D. students who entered the
graduate teaching profession, and their students who entered the
graduate teaching profession, and so on. Each school cited is either
the school at which the person now teaches or the last school at
which the person taught before moving on to other pursuits.
Postdoctoral associates, undergraduate associates, and nonteaching
descendants are omitted. The compilation was made by Paul
Beckwith, Dave Coleman, Stan Crouch, Scott Goode, Alex
Scheeline, Jim Winefordner, and others.
Howard V.
Malmstadt
J. D. Winefordner
Florida
Clifford Toren
South Alabama
Harry L. Pardue
Purdue
John P. Waiters
St. Olaf
Williard Harrison
Virginia
Ed Piepmeier
Oregon State
Linda J. Cline Love
Seton Hall
S. R. Crouch
Michigan State
Gary Hieftje
Indiana
Gary Horlick
Alberta
Ramon Barnes
Massachusetts
Kuang-Pang Li
Lowell
Kelly O'Keefe
Colorado State
Brian Renoe
Virginia
T. Hadjiioannou
Athens
M. Bonner Denton
Arizona
Collen Delaney
Univ. Washington
James Avery
Colorado
Jim DeFreese
Kansas
Alan Wu
Texas
Melton Bryant
Georgia
I Edward P. C. Lai
Carlton
obert Green
Howard/.atz Arkansas
Ohio Univ.
Robertwyoming
J' Hurtubise
={ Lloyd Remington
North Carolina-Ashville
,.' Hwa'rd Williams
Southern Mississippi
= Tom Carnes
( J. Do Winefordner
.,.Th O'H
...( Andrew Zander
Florida Maryland Cleveland State
Arizona State West Virginia
,( Th Vickers th W. Busch
Florida State Baylor
. S.Y. Su
Virginia Commonwealth
{ c,,o0ovo,,,oo
Houston
Frank Planke,
--( Ed Heithmar
Pittsburgh New Orleans
O.,o,,.rO
Marquette
George Cembrowski
Pennsylvania
Clifford Toron James Eo Davis
South Alabama Washington Univ,
R. Neill Carey
Wisconsin
HarryL" Pardue
South Carolina
Purdue
Stanley Doming
Houston
Steven Morgan
,. Richard Sacks
Texas New Mexico State
Michigan
Joel
Vermont
Goldberg
,. St
Eastern Michigan
.DavidCol
Jhn P' Waiters
"St.
Olaf
Wayne State
.Alexander Scheeline
Illinois
,,PaulFa orth
Brigham Young
,ohoo,e.,,
North Carolina
Samuel Matthews
Johns Hopkins
Kenneth Marcus
Clemson
Williard Harrison Carlos Bruhn
Virg!nia Santiago
Kenneth Mess
Franklin Marshall
Yehia M, A.'Slii
..scott Goode San,a' Univ, (Yemen).]
South Carolina James Deavor
Charleston
Michigan State Oregon State McGill
,. Akbar Montaser
George Washington
, James Holler
Kentucky
K.C. Lin
National Taiwan
o.,.o,,
U. of the Andes
North Carolina State VirginiaPolytechnic
N. C. Clampitt
Luther College
..David Honigs
Washington
Texas A&M Regio (Mexico)
GaryHorlick
..( Michael Blades
Alberta Bdtish Columbia
M.
BonnerAdzonaDenton KansasRbertstateFry
199
Howard Malmstadt
place, the FACSS conference became notjust a tribute to
the accomplishments of Howard Malmstadt, but also a
celebration of the man's spirit. Many of the direct
descendants shown in the family-tree, as well as many
professional colleagues who have worked with Malmstadt
through the years, attended the conference. As acquain-
tanceships were renewed and old times recalled, the
participants shared in the themes of respect and enthu-
siasm for Malmstadt and his work.
The programme also featured three half-day symposia,
each of which was conducted by former students and
colleagues of Malmstadt. These symposia, which
explored analytical trends and issues ofwider scope, were
as follows:
'Emerging concepts in instrumentation and education,
'New concepts in spectrochemical analysis', and
'Advances in biotechnological.and clinical methodology'.
Malmstadt closed the first symposium with an address
entitled 'Technology, education, and communities in
need'. In this moving oration, he explored profound
issues ofchemistry in a social context, drawing on his own
appropriate experience. Malmstadt envisioned an
academic curriculum that would recognize the needs of
communities and provide a framework for satisfying
those needs through the course ofacademic study. In this
curriculum, the main objectives of education would be
ever present, as problems involving energy, the environ-
ment, health, sanitation, and engineering would be dealt
with literally. Problems would be identified, the skills
required to solve them would be determined, and
students would be trained appropriately. Students would
work in the field so that their experience would result in a
mix of the practical and empirical aspects of problem
solving together with the ideal and theoretical aspects.
In his role as a teaching professor, Howard Malmstadt
insisted that his students develop and maintain a broad
perspective on their discipline. As Gary Hieftje put it, 'He
taught that students should not lose sight of their
heritage, that is, the chemistry'. Malmstadt's remarks at
the FACSS conference revealed an insistence on an even
broader perspective. He now calls upon chemistry
students not only to be aware of the wide base of
knowledge and the history that surround their work, but
also to recognize the responsibilities and possibilities
inherent in the relationship between the science and
society.
At the Pacific and Asia Christian University, Howard
Malmstadt now contributes his knowledge and talents to
a programme that is meant to help people living in the
Pacific Basin area. He has come full circle since that time,
some 40 years ago, when he served as a radar officer in the
Pacific theatre and wasjust starting his career in science.
In the intervening years, Malmstadt has worked with
countless young scientists, instilling in them good values
while pointing them in the direction ofgood science. This
man of patience and brilliance and vision has been, and
continues to be, an inspiration.
EDITOR'S NOTE
Howard Malmstadt is certainly unique in analytical chemistry, but most ofall he is quick also to recognize the
value ofwork carried out by other groups. When he visited us at the Laboratory ofthe Government Chemist in
the early 1970s, everyone was impressed by the work he had achieved, particularly in the education ofchemists
in automation, especially electronics training. Howard had made it simple, and by giving a 'hands-on' approach
was able to get chemists confident with the new facilities available. But in his own style, Professor Malmstadt
was quick to see the value of the work done at LGC, particularly with respect to sample handling and
preparation. On a visit to the USA, I remember being introduced to Howard's group at the University of
Urbana-Champaign and was very flattered by his comments on the work done by LGC. Before I could get over
this I was almost taken aback by the confidence ofthis group, who after only five minutes or so ofintroduction
were throwing questions at this lecturer. They were keen to know more details about the work I was describing.
Howard's training had made the group very enthusiastic and also keen to see how other people's work could be
used in their own environment. Howard and all ofhis group were keen to recognize the reality ofanalysis and to
deal with real-world sampling situations.
Howard was also very instrumental in helping me get theJournaloffthe ground. A quick look at the membership
ofthe editorial board will show this. I remember that he told me it will take a long time for people in America to
recognize the value of automation rather than simply computerization. By his enthusiasm and help, we have
persevered and we take JAC into the next decade as a well-respected journal.
Thank you Howard for your help and encouragement. Long may we continue to have your valued support.
Peter Stockwell
200

				

